# The Positive Impact of Bariatric Surgery on Vascular Health

**DOI:** 10.7759/cureus.57586

**Published:** 2024-04-04

**Authors:** Fazal Dalal, Monique Hassan, Robert J Widmer

**Affiliations:** 1 Department of Internal Medicine, Baylor Scott & White Medical Center - Temple, Temple, USA; 2 Department of Surgery and Bariatric Surgery, Baylor Scott & White Medical Center - Temple, Temple, USA; 3 Department of Cardiology, Baylor Scott & White Medical Center - Temple, Temple, USA

**Keywords:** weight loss and obesity, vascular function, sleep apnea, endothelial function, bariatric surgery

## Abstract

Background: Obesity is one of the most prevalent medical conditions in the Western world. There are many risk factors associated with obesity, including cardiovascular and pulmonary risk. Vascular health is not studied in obese patients, and whether obesity has an adverse effect on vascular health in these patients remains unknown.

Objective: The first objective is to find a correlation between vascular health and obesity and whether obesity can be classified as a risk factor for vascular health. The second objective is to see if weight loss leads to an improvement in vascular health in patients.

Methods: The study was conducted with pre- and post-surgical methods at Baylor Scott & White (BSWH) Medical Center, Temple, Texas, USA. Ten patients were approached, consented, and prepared to obtain baseline values through WatchPAT and EndoPAT devices prior to their bariatric surgery. Values obtained include their initial weight, respiratory disturbance index, apnea-hypopnea index, oxygen desaturation index, and degree of endothelial dysfunction via the EndoPAT device. Post-surgery, these values were obtained again and compared using Wilcoxon non-parametric analyses with a level of significance at p < 0.05.

Results: Our study results demonstrate a correlation between obesity and vascular health as endothelial dysfunction is widely seen. In our patients, after bariatric surgery, we saw a significant weight change (31.2% +11.2, p < 0.0001). There was a significant degree of endothelial function improvement after the weight loss (31.2% +34.7, p < 0.04).

Conclusion: Our results indicate that there is a correlation between obesity and vascular health, which also correlates with cardiovascular risk. There is a significant reduction in endothelial dysfunction after weight loss. We believe that obesity is a risk factor for vascular health outcomes.

## Introduction

Obesity is defined as a body mass index (BMI) of over 30.0 and has become one of the most prevalent conditions in the world. Current statistics indicate that the current rate of obesity is over 30% for both men and women, growing at a rate of 8% in the past 35 years, and has a disparate impact on different racial groups [1]. Obesity is typically a primary disease caused by calorie intake/expenditure imbalances and carries with it a high rate of progression to other chronic diseases, such as type 2 diabetes mellitus, coronary artery disease (CAD), hypertension (HTN), hyperlipidemia, degenerative joint disease, obstructive sleep apnea (OSA), dementia, and stroke [2,3]. All the aforementioned conditions usually consist of an underlying inflammatory condition, all of which are linked to peripheral endothelial dysfunction (PED) [4-7].

PED, defined as an inappropriate vasomotor response to physiologic and pharmacologic stress, is considered a basis for vascular risk factors as it may represent a preclinical atheromatous state [4-7]. Moreover, PED is a risk factor for the development of CAD, hypertension, stroke, and dementia [8-11]. Recent studies attempting to ascertain the response of the microvasculature to surgically induced weight loss have shown no impact; however, the role of the peripheral microcirculation remains unknown [12]. PED is involved in both early and late mechanisms of atherosclerosis [13-16] and is a strong predictor of cardiovascular mortality [7,17]. 

Impairment of endothelial function is related to the severity of obesity [18-19], and endothelial dysfunction appears to be reversible with weight loss [20]. While obesity is considered a risk factor for the presence of PED and progression to CAD and cerebrovascular disease, little is known about the relationship between obesity and PED [21,22]. To date, few studies have evaluated the relationship between weight loss and endothelial function [12,19-20].

Here, we present a pilot study whereby we measure peripheral endothelial function and measures of obstructive sleep apnea before and after bariatric surgery to ascertain data on the potential association of weight loss to concomitant improvement in vascular and sleep health.

## Materials and methods

This was an institutionally approved and funded study through the Baylor Scott & White (BSWH), Temple, Texas Institutional Review Board (IRB# 021-003). The participants were patients referred for bariatric surgery and currently undergoing workup for eligibility at BSWH, Temple, Texas. This was a pilot study between the cardiology and bariatric surgery divisions to see if this advanced testing would yield any new data or patient/quality efficiencies. Ten patients were enrolled as a part of a pilot trial with hopes to roll out to all bariatric patients if successful. The patients were approached, consented, and enrolled during their cardiovascular clearance appointment. All workups were standard for the bariatric surgery program except for vascular reactivity testing. Obstructive sleep apnea (OSA) testing was standard, and WatchPAT testing was an additional test on top of the usual OSA evaluation.

Baseline and six-month postoperative demographic and anthropomorphic variables were obtained via the National Surgical Quality Improvement Program (NSQIP) database, which the Surgical Department at BSWH Central TX contributes. These data were then matched with EndoPAT and WatchPAT data as obtained below.

FDA-approved device EndoPAT was performed as described previously [7], including manuscripts by the principal investigator [23] who has no financial or patent interest in the study device. EndoPAT is a device that assesses endothelial function. EndoPAT testing was conducted in a quiet thermo-neutral room in the CV division, desk 1H, by a trained study technician using a standard operating procedure reviewed annually. The participants rested comfortably in a reclining chair with feet elevated parallel to the heart. Baseline blood pressure (BP) was obtained with an electronic sphygmomanometer, and an initial EndoPAT reading was to be obtained for five minutes at rest. The BP cuff was then inflated to at least 50 mmHg above baseline systolic pressure, and vascular occlusion (confirmed with device monitoring) was maintained for five minutes. The cuff was then deflated, and the continuous reading of arterial hyperemia was obtained for another five minutes. The overall response was converted into a continuous variable and then a logarithmic transformation score for uniformity.

Sleep apnea was assessed via the WatchPAT100 (Itamar Medical LTD, Caesarea, Israel) before and six months after bariatric surgery in a fashion similar to that previously described [24]. This is a self-containing device worn around the wrist. Two finger probes extend from the main body of the device; one is the optico-pneumatic sensor that detects the PAT signal, and the other measures arterial oxygen saturation. The body of the device also contains an actigraphy (three-axis accelerometer for detection of limb activity), which is used. The WatchPAT continuously records four physiologic signals throughout the night (PAT, oxygen saturation, heart rate, and actigraphy). These data are then analyzed with an automated computerized algorithm that calculates the frequency of respiratory events per hour of actigraphy-determined sleep. Respirations were detected during sleep (per actigraphy) using a combination of PAT signal attenuation, desaturation on pulse oximetry, and changes in heart rate. A respiratory event will be noted if one of three criteria is met: (1) a 30% or greater PAT amplitude reduction together with a pulse rate acceleration of 10%, (2) a 30% or greater PAT amplitude reduction together with a 3% oxyhemoglobin desaturation, or (3) a 4% oxyhemoglobin desaturation. The primary measure of OSA will include the apnea-hypopnea index (AHI), which is an aggregation of the measures listed above and assessed on a scale of 1-15 with a value over 5 indicative of OSA. Measures, such as the respiratory disturbance index (RDI) and oxygen desaturation index (ODI), will also be measured and assessed. All patients in the study were diagnosed with sleep apnea and were under the BSWH Sleep Medicine Clinic's care.

Descriptive statistics summarizing the characteristics of the study population were calculated for demographic, behavioral, anthropometric, medical comorbid, and endothelial function measures. The distributions of continuous measures were inspected, and variables with skewed distributions were summarized with the median and first and third quartiles; those with near-normal distributions were summarized with the mean and standard deviation. Discrete variables were summarized with frequencies and percentages. Percent changes were calculated as the difference from the baseline to post-surgical divided by the baseline and then multiplied by 100 for each individual patient and then averages among the cohort. Group differences were tested using either Student’s paired t-test, the Wilcoxon sign-rank test, or McNemar’s test according to the data type and distribution. All tests were two-tailed with a type I error rate of 0.05 and conducted on an intention-to-treat basis.

## Results

Our baseline demographic data includes age, female sex, race, BMI, smoking status, diabetes mellitus, OSA, GERD, prior cardiovascular disease, hypertension, and hyperlipidemia (Table 1). Based on the statistical analysis, weight change in the pre-surgical and post-surgical cohorts was statistically significant (294.0 ± 69.4 lbs to 198.4 ± 47.4 lbs, -31.2% ± 11.2, p < 0.0001) (Table 2). EndoPAT measurements in the pre-surgical and post-surgical patients were also statistically significant (0.63 ± 0.17 to 0.79 ± 0.25, -31.2% ± 34.7, p = 0.04). The augmentation index, WatchPAT RDI, AHI, ODI, and O_2_ desaturation were not statistically significant, as shown in Table 2. Percent change for each category was calculated for each category showing significance in weight and Ln EndoPAT (Table 2).

**Table 1:** Baseline demographics Baseline demographics for the participants at enrollment. Continuous variables are listed as mean ± standard deviation (SD), and binary variables are listed as the number/counts (N) per total cohort and percentages (%).

Baseline demographics	Value
Age, years (mean+/-SD)	57.5±9.9
Female sex (N, %)	8/10 (80%)
White race (N, %)	8/10 (80%)
Highest BMI, kg/m^2 ^(mean+/-SD)	50.0±10.5
Current smokers (N, %)	0/10 (0%)
Diabetes (N, %)	5/10 (50%)
Obstructive sleep apnea (N, %)	9/10 (90%)
Gastroesophageal reflux disease (N, %)	2/10 (20%)
Prior cardiovascular disease (N, %)	0/10 (0%)
Hypertension (N, %)	7/10 (7/10)
Hyperlipidemia (N, %)	5/10 (50%)
Dyspnea at baseline (N, %)	6/10 (60%)

**Table 2:** Results Baseline and six-month post-surgical vascular and sleep apnea data. Continuous variables are listed as mean ± standard deviation (SD), and binary variables are listed as the number/counts (N) per total cohort and percentages (%). Percent changes were calculated for each individual patient as the difference from the baseline to post-surgical divided by the baseline and then multiplied by 100. Statistical significance for paired comparisons is considered <0.05. RDI: respiratory disturbance index, AHI: apnea-hypopnea index; ODI: oxygen desaturation index

	Pre-surgical	Post-surgical	% change	P-value
Weight, lbs (n = 10)	294.0 ± 69.4	198.4 ± 47.4	-31.2 ± 11.2	<0.0001*
Ln EndoPAT (n = 6)	0.63 ± 0.17	0.79 ± 0.25	+31.2 ± 34.7	0.04*
Augmentation index (n = 4)	0.06 ± 0.13	0.09 ± 00.32	+160.9 ± 490.8	0.28
WatchPAT RDI (n = 4)	34.2 ± 20.8	22.9 ± 11.0	-6.7 ± 54.5	0.41
WatchPAT AHI 3% (n = 4)	32.3 ± 20.8	20.7 ± 10.6	-13.2 ± 48.8	0.31
WatchPAT ODI (n = 4)	19.5 ± 17.8	10.2 ± 8.5	+19.4 ± 142.9	0.40
WatchPAT O_2_ Desat (n = 6)	82.3 ± 76.2	37.5 ± 21.5	+62.6 ± 179.9	0.22

## Discussion

In this initial pilot study of 10 patients undergoing surgical weight loss, we show a concomitant loss in weight and improvement in peripheral endothelial function without other improvements in vascular or pulmonary variables. These data underscore a potential mechanism for the early changes in larger cardiovascular improvements seen with bariatric surgery and might identify early tracking targets to ensure that patients will be positive cardiovascular responders to weight loss surgery.

A recent study shows that 81% of Americans consider obesity to be a leading health problem and that nearly all believe that, by itself, it can contribute to early death [25]. In this initial pilot study of 10 patients undergoing surgical weight loss, we show a concomitant loss in weight and improvement in peripheral endothelial function without other improvements in vascular or pulmonary variables. These data underscore a potential mechanism for the early changes in larger cardiovascular improvements seen with bariatric surgery and might identify early tracking targets to ensure patients are positive cardiovascular responders to weight loss surgery. 

Our pilot study conducted at BSWH Medical Center shows how endothelial dysfunction is affected in patients with morbid obesity treated with weight loss surgery. On a biochemical level, the adipose tissue releases inflammatory cytokines (TNFa and IL-6), along with nitric oxide bioavailability, insulin resistance, and oxidized low-density lipoprotein (LDL), which all play a key role in endothelin dysfunction in obese patients [26]. The increased risk of cardiovascular diseases with morbid obesity is evident, but very little evidence exists on vascular health. EndoPAT is a device that allows us to check vasculature in patients further affirming that obesity causes endothelial dysfunction that improves with weight loss.

Our affirmative results are substantial information that allows us to learn about how obesity affects our vascular health. This in turn has a direct correlation with cardiovascular diseases, which is severely impacted in morbid obese patients. OSA in severely morbid obese patients plays a significant role in cardiovascular health, but it may be a “lagging” indicator of obesity and poor vascular health [27]. Our results suggest that losing weight in patients is paramount to their cardiovascular health with the first improvement being an improvement in vascular reactivity within six months of surgery and prior to any substantial improvements in OSA. In obese patients, their vasculature loses its physiological properties, such as the tendency to promote vasodilation, fibrinolysis, and anti-aggregation [28]. With such dysfunction, they are more prone to vascular diseases, which leads to poor health outcomes in these patients. While obesity is a common issue in the USA and clinicians are actively pursuing to treat this condition, we now know how it affects other parts of the body that can accelerate a downward spiral on their health. Clinicians should also think about how obesity affects almost every part of their body.

A limitation of our study is the sample size. Our sample size is small, primarily due to the lack of recruitment opportunities in our trial. While our data are significant, we believe that having a larger sample size would yield better results. Another limitation is not being able to study individuals who underwent weight loss through diet and exercise. Our study only includes candidates who underwent bariatric surgery and whose endothelial dysfunction improved. It is unclear, however, if patients who underwent weight loss through other means would have similar results. A study in the future could be conducted comparing the degree of improvement in vascular health among patients who lose weight with bariatric surgery compared to patients who lose weight with a diet/exercise regimen.

Our pilot study was conducted to evaluate the correlation between obesity and vascular health. Results show that there is a significant correlation between obesity and endothelial dysfunction. The EndoPAT device was able to tell us the degree of endothelial dysfunction [29]. After post-surgical weight loss, the endothelial dysfunction improved and so did their vascular health. This plays a key role in a patient’s risk reduction for cardiovascular disease. As obesity is on a rapid rise in the USA and affecting many people, these findings would help clinicians better understand obesity’s impact on their patients. Early intervention in patients with obesity is paramount to their health by starting on an appropriate diet/exercise regimen. We believe that patients who have morbid obesity should undergo vascular testing with devices, such as WatchPAT and EndoPAT, to get a baseline understanding of their vascular health, track progress with weight loss, and make treatment plans accordingly. A future study regarding such practices would be interesting to see in the overall health and survival of obese patients.

## Conclusions

Our study indicates the direct correlation between weight loss and vascular health. The weight loss was significant, and the vascular health, as measured by EndoPAT, showed significant improvement following surgery. While a larger sample size would improve the results further, we believe that endothelial dysfunction is seen in morbidly obese patients, and clinicians should add that as a further risk factor for such patients.
